# Force sharing and other collaborative strategies in a dyadic force perception task

**DOI:** 10.1371/journal.pone.0192754

**Published:** 2018-02-23

**Authors:** Fabio Tatti, Gabriel Baud-Bovy

**Affiliations:** 1 Robotics, Brain and Cognitive Sciences Department, Istituto Italiano di Tecnologia, Genoa, Italy; 2 Faculty of Psychology, Università Vita-Salute San Raffaele, Milan, Italy; 3 Experimental Psychology Unit, IRCCS San Raffaele Scientific Institute, Milan, Italy; University of Akron, UNITED STATES

## Abstract

When several persons perform a physical task jointly, such as transporting an object together, the interaction force that each person experiences is the sum of the forces applied by all other persons on the same object. Therefore, there is a fundamental ambiguity about the origin of the force that each person experiences. This study investigated the ability of a dyad (two persons) to identify the direction of a small force produced by a haptic device and applied to a jointly held object. In this particular task, the dyad might split the force produced by the haptic device (the external force) in an infinite number of ways, depending on how the two partners interacted physically. A major objective of this study was to understand how the two partners coordinated their action to perceive the direction of the third force that was applied to the jointly held object. This study included a condition where each participant responded independently and another one where the two participants had to agree upon a single negotiated response. The results showed a broad range of behaviors. In general, the external force was not split in a way that would maximize the joint performance. In fact, the external force was often split very unequally, leaving one person without information about the external force. However, the performance was better than expected in this case, which led to the discovery of an unanticipated strategy whereby the person who took all the force transmitted this information to the partner by moving the jointly held object. When the dyad could negotiate the response, we found that the participant with less force information tended to switch his or her response more often.

## Introduction

Successful collaboration between two partners requires coordination. In tasks where two people manipulate an object or a tool collaboratively, each partner needs to know what the other is doing and anticipate the consequences of the action [1]. In joint tasks involving physical interaction such as moving a table together, the haptic modality could provide additional information about what the partner is doing or intends to do, which might also help coordinate action (e.g. [2,3]). While several studies have suggested that the haptic channel plays an important role in coordinating action (reviews in [4,5]), previous studies have not directly investigated the perception of the forces applied to an object in situations involving more than two agents. In these situations, it is important to note that the force that each agent experiences is the sum of all the other forces applied to the object. For this reason, it is difficult to infer the individual contributions of the other agents from the interaction force when more than two agents apply a force on the same object. For instance, if two people pull on a rope against a third person, this person cannot know the particular force that each person on the other side is exerting.

In this study, we consider a situation involving three agents—two people and a haptic device—who apply a force to the same object, a bar mounted on end-effector of the haptic device. The two people are facing each other and the object moves along a line in between the two people. The two people need to keep the object immobile in between them while the haptic device is producing a small force toward one of them. In the following, we refer to the force produced by the device as the external force and to the forces produced and/or experienced by the dyad as the interaction forces. In this quasi-static situation, the sum of the two interaction forces must balance the external force exactly. The task for each person is to make a judgment on the direction of the external force, i.e. the force applied by the device on the object. Because the interaction force that each person experiences depends in part on what the other person is doing, the interaction force provides ambiguous information about the external force.

The general objective of this study is to investigate how two people might coordinate their action in order to find out how much force a third agent is applying to the same object. In this situation, the two people can balance the external force in an infinite number of ways. The particular way the two people will oppose the external force will determine the interaction force that each person experiences. In other words, the information that the interaction forces provide about the external force depends on the dyad’s motor behavior. The first objective of this study is, therefore, to find out how the dyad splits the external force, and how this relates to the individual performances in this task. The second objective is to test a hypothesis that the dyads might coordinate their motor behavior to maximize their joint performance by splitting the external force according to their relative force sensitivity. We call this last possibility optimal force sharing.

One novelty of this study is that the participants must coordinate their action at the physical level to perform a perceptual task. In contrast, previous studies on joint physical action have investigated the performance of dyads in motor tasks (reviews in [4,5]). As noted above, the problem faced by the participants in this task is that the interaction forces provide ambiguous information about the external force. In this respect, it is noteworthy that sensory systems often have to deal with ambiguous stimuli. For example, in the so-called “aperture problem”, the visual system is presented with a stimulus that does not allow the observer to know with certainty the direction and/or velocity of motion of the visual stimulus [6]. In this case, the ambiguity about the velocity can be resolved by making assumptions about the direction of movement of the stimulus for example [6,7]. Similarly, the ambiguity in our task could be resolved if the two partners make assumptions about the force produced by the other partner. Another possibility is that the two people adopt motor behaviors such that the direction of the interaction forces reflects the direction of the external force.

Previous studies have shown that observers can infer the hidden properties of an object such as its weight by simply watching a person interact with the object (e.g. [8,9]). Humans also have the ability to use very subtle visual cues to read other people’s actions and intentions (reviews in [10,11]). Numerous studies have shown that these abilities play an important role in joint action (e.g. [12]). While these studies have focused on the contribution of visual information, one might surmise that humans also have the ability to coordinate their behavior to gain information about each other’s action and/or the environment through the haptic modality when interacting physically.

To clear up a possible confusion about the purpose of this study, it is important to note that we do not claim that dyads routinely estimate the various forces and torques that are applied to an object when performing joint physical actions, such as moving a table together. They are probably more focused on the movement of the object than on the perceived forces in these motor tasks. Still, it might be useful if not necessary in some contexts to be able to untangle the different force contributions. For example, pilots flying aircrafts with a traditional manual system, where the captain’s and co-pilot’s control wheels are mechanically coupled to each other and to the aircraft’s control surfaces [13], might need to untangle the force coming from those control surfaces from that of the other pilot. Similarly, in robot-assisted training, guidance forces provided by a (real or virtual) teacher or therapist are often mixed with task forces, such as viscous or elastic force fields and the participant needs to untangle the two components in order to correctly interpret the guidance provided [14].

The paper is organized as follows. First, we define the task and experimental procedure in the next section. In the main experimental condition, each member of the dyad must respond independently. In another condition that aimed at understanding whether the dyad’s behavior or the participants’ performance would change if the dyad could communicate, the two members had to agree on a common response. Finally, we also measured the capacity of each person to perceive the direction of a force separately. In the section after, we formally present the optimal force-sharing model. In the results section, we describe the force sharing strategies adopted by the dyads and compare their performance to the model predictions. In general, we found that the dyads did not split the external force in a manner that maximized joint performance. Surprisingly, we also found that individuals who did not get any information about the external force in some dyads out-performed the model’s predictions and chance level. This observation led us to identify an unanticipated response strategy based on positional information, which explains these individuals’ level of performance. Finally, we also show that the dyads outperform the model predictions when they must agree on the response.

## Methods

### Participants

32 people participated in the experiment (23 females, 9 males. Mean (±SD) age = 26.22 ± 4.48 years). They were divided into 16 dyads. All participants were right handed as assessed by the Edinburgh Handedness Inventory [15] and declared no known history of neurological disorders. Participants were recruited from a mailing list of volunteers and received compensation for their participation in the experiment. The protocol was approved by the local ethical committee (Azienda Sanitaria Locale 3, Genova) and conducted according to the principles expressed in the Declaration of Helsinki. All participants signed an informed consent form prior to testing.

After the test, each participant was asked to complete the Interpersonal Reactivity Index questionnaire [16] and to respond to a questionnaire with general questions about the experiment and the participant’s experience with haptics.

### Experimental setup

**Fig 1:**
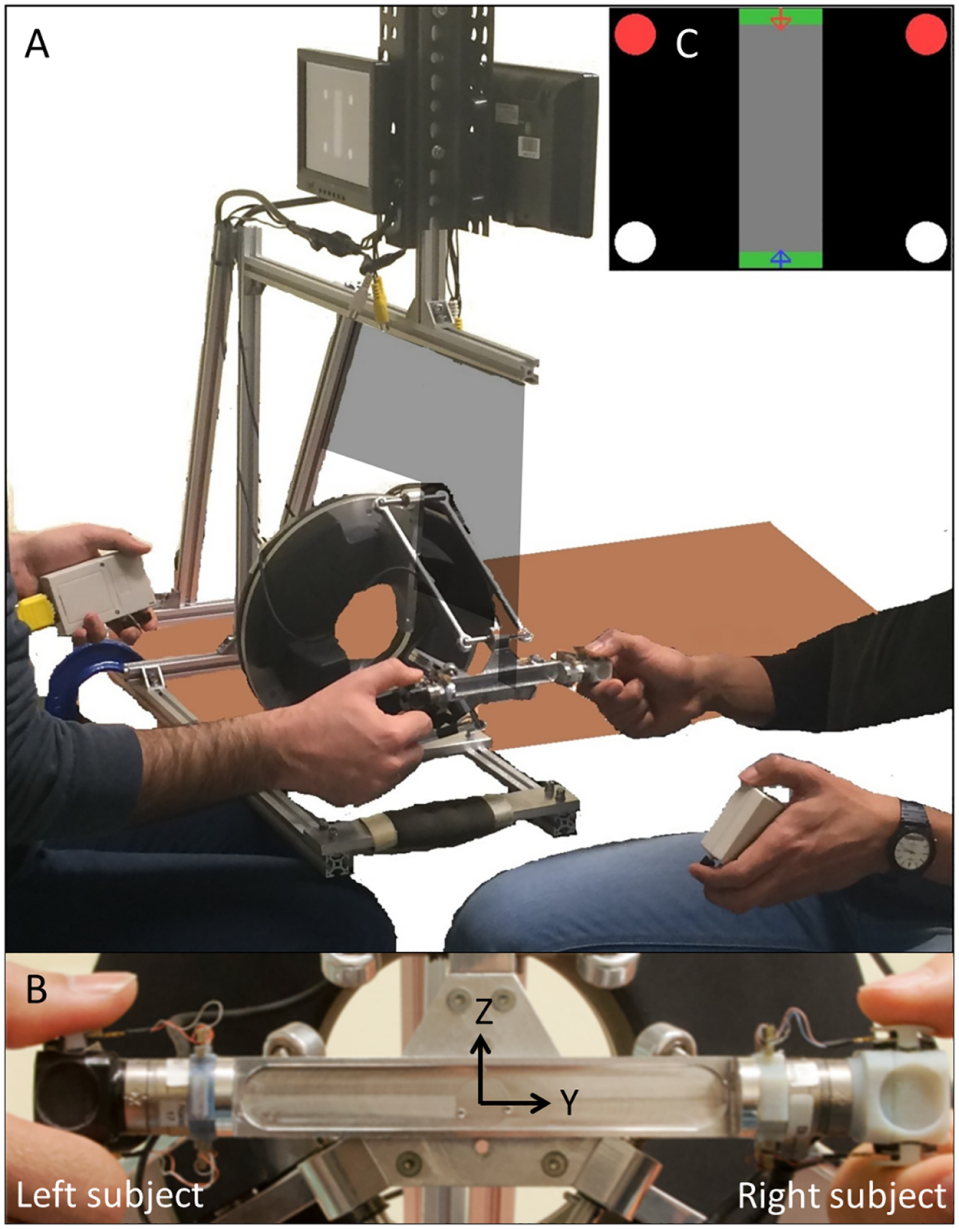
Experimental setup. ***A***: Overview of the setup. A board separated two participants to prevent them from viewing each other’s hand. ***B***: Detail of the end-effector showing the two ATI force sensors measuring the interaction forces and the force pressure sensors measuring the grip force. ***C***: Visual feedback provided on the screen on the grip force and the response. The two disks on the left and right sides correspond to the two possible responses of the two participants. The red disks indicate the responses of the participants (which coincide in this example). The response of the other participant was shown only in the joint negotiated condition (BN). The two arrows in the central part provided visual feedback about the grip force. The tips of the arrows had to be kept within the green area.

The experimental setup was composed of an Omega.3 haptic device (Force Dimension, Nyon, Switzerland) with an end-effector customized for this experiment, two 7” monitors, and two boxes with buttons used to collect the participants’ responses. Fig 1A provides an overall view of the setup.

The haptic device was placed in between the seated participants who faced each other and grasped the end-effector of the haptic device with their right hands (key grasp). Their chairs were positioned so that the device end-effector was aligned with each participant’s right shoulder and the chairs’ height was adjusted so that participants could interact with the device while keeping their right arms in a 90° posture. The height and position of the monitors were adjusted to be comfortable for each participant. A panel prevented participants from seeing each other, including their right hands.

Fig 1B shows the custom end-effector in greater detail. It is composed of an aluminum bar whose ends have been equipped with two Nano 17 Force-Torque sensors (ATI, Apex, NC, USA), and two cubic user interfaces mounted on the tool side of each FT sensor. Each user interface houses two Force Sensing Resistors (FSR^™^ 400 Interlink Electronics, Camarillo, CA, USA) used to monitor the grip force.

To avoid ambiguity in the description of the task and stimuli, we define a global frame of reference centered on the haptic device with the Y axis aligned with the participants and the Z axis aligned vertically (see Fig 1B). The bar mounted on the device and grasped at each extremity by a participant could move along the Y axis freely. The movements of the bar in the other directions (±X or ±Z) were limited by a stiff elastic force field (stiffness: 500 N/m). The external force *f*_*ext*_ produced by the device and the interaction forces *f*_*R*_ and *f*_*L*_ produced by the participants are all aligned with the Y axis. By definition, the Y+ axis points toward the right participant and the Y- axis points toward the left participant.

The system was controlled by a software program running on a PC with a Windows 7 OS. A 16-bit data acquisition card (PCI-6225, National Instruments, Austin, TX, USA) was used to acquire data from the sensors. The software was written in C++ using Visual Studio 2008. The Omega.3 was controlled by using the Force Dimension DHD API, and the NI-DAQmx C API was used to interface with the data acquisition card.

### Experimental procedure

Each trial began by displaying a written instruction that reminded the participants to position the device at the center of the workspace (midway between them). A visual cue informed participants about the correctness of their position. Once the position was correct, the experimenter started the trial and the device applied a force *f*_*ext*_ directed towards one of the two participants. The force applied by the device slowly ramped up from zero to the target value over a 5-second ramp period. An auditory signal indicated the end of the ramp period and the force remained constant thereafter, until the participants’ responses were collected. We instructed the participants to hold the device at this initial central position throughout the trial and to press one of the buttons on the response box to indicate the perceived direction of the force applied by the haptic device, i.e. toward them or toward their partner. The responses of the participants were recoded to indicate the perceived direction of the external force in the global reference frame (i.e., toward the left “Y-” or right “Y+”participant, see Fig 1B).

During the trial, the display provided visual feedback about the grip force (see Fig 1C). Two arrows indicated the force recorded from the top and bottom FSR sensors mounted on each handle held by the participant. Each participant had to keep the tip of the arrow within the green area, which corresponded to a grip force level in the 0 to 1 N range. On the left side of the grip force indicator, a pair of “visual buttons” provided visual feedback about the participant’s own response at the end of each trial. Another pair of visual buttons was used to inform each participant about their partner’s response in the “negotiated” response condition.

### Experimental conditions

Four experimental conditions were included in the experiment: two individual (L and R) and two joint (BI and BN) conditions. In the individual conditions, the left (L) and the right (R) participant performed the task alone, while the other person did not touch the device. In the joint conditions, the two participants grasped the device together: In the “both independent” (BI) condition, the two participants provided their response independently via their response box; in the “both negotiated” (BN) condition, the two participants had to agree on a common response. The joint response in the BN condition was obtained in the following manner: first, each participant responded separately via the response box. Once both responses were produced, the two participants received a visual feedback of the partner’s response (see Fig 1C). If the two responses differed, the two participants had to respond again. The process was reiterated until the two responses coincided.

The magnitude of the force produced by the haptic device ranged from 0 N to 1.5N (see Table 1). The magnitudes of the force stimuli were greater in the joint conditions than in the individual ones because we expected the external force to be split between the two participants in these conditions. Therefore, we increased the force magnitudes in the joint conditions to try to maintain the performance level in the individual and joint conditions. We used smaller force stimuli after the first 8 dyads, because we found that the largest force values were too easy to perceive and therefore not very informative. Note that the results do not depend on the exact force levels used in the experiment, which we selected to allow us to estimate the sensory thresholds and measure the performance level at 0.5 N in all conditions (see Data analysis).

**Table 1:** External force (f_E_) in [N] used as stimulus.

Condition	Dyads 1–8	Dyads 9–16
Single (L and R)	0.0, ±0.15, ±0.3, ±0.45	0.0, ±0.08, ±0.16, ±0.24
Joint (BI and BN)	0.0 ±0.5, ±1.0, ±1.5	0.00, ±0.3, ±0.6, ±0.9

The four experimental conditions were tested during the same session. The session was divided into 40 blocks of 7 trials, which corresponded to the 7 force levels presented in a random order. The condition was fixed within one block but the order of the blocks was randomized. The total number of trials for each dyad was 7 force levels × 4 conditions × 10 repetitions = 280 trials.

### Haptic device control

The rendered force was computed at 1KHz. Because the force actually rendered by the haptic device does not generally reflect the one commanded, we used a force-feedback closed-loop scheme to improve the quality of force rendering along the unconstrained direction (Y axis):

$$f_{y}\;=\;f_{d}-k_{p}f_{e}\;=\;f_{d}-k_{p}(f_{m}-f_{d})$$

where *f*_y_ is the force commanded to the Omega.3 along the Y axis, *f*_d_ is the desired force, *f*_m_ is the measured force, *f*_e_ is the force error and *k*_*p*_ is the force feedback gain. The desired force is the external force (i.e. *f*_d_ = *f*_ext_) and the measured force *f*_m_ = *f*_R_ + *f*_L_ corresponds to the sum of the two interaction forces measured by the force sensors mounted on the handles (see Fig 1B). The interaction forces were sampled at 15 kHz and the last 15 samples were used to compute the interaction force used in the haptic control loop.

The closed loop control guarantees that the desired external force corresponds to the sum of the interaction forces, i.e. *f*_ext_ = *f*_R_ + *f*_L_, throughout the trial, independently from the device’s motion, inertial and friction forces. In this study, the average (±SD) RMS force error *f*_e_ between the measured and desired external force was 0.03±0.01N. In 5% of the trials, the haptic interaction exhibited a period of instability, which yielded an RMS error larger than 0.05 N. 62% of these trials were performed by 4 participants when interacting alone with the setup. Interestingly, the instability occurred much less frequently in the joint conditions, where these participants interacted with their partners. A more detailed discussion of the closed-loop controller can be found in [17].

The forces along the X and Z directions corresponded to an elastic force field that constrained the end-effector movement along a line parallel to the Y axis and passing through x_d_ and z_d_ positions:

$$\begin{array}{ll} f_{x} & =k_{s}\left(x_{d}-x\right)\\ f_{z} & =k_{s}\left(z_{d}-z\right) \end{array}$$

where *x* and *z* are the current position of the end effector and *k*_*s*_ = 500 N/m is the stiffness of the elastic force field.

## Data analysis and modeling

All analyses and modeling were performed using R and its contributed packages [18]. Statistical analyses are described in the result sections.

### Force sharing

In our task, the haptic device and the two participants apply a force on the rigid bar. In the following, we shall define the interaction forces *f*_L_ and *f*_R_ as the forces experienced by the left and right participants respectively. The interaction forces experienced during the task correspond to the applied forces but have the opposite sign (e.g. if a participant produces a force towards +Y, he or she will experience a reaction force towards -Y). In the quasi-static condition corresponding to the task, the forces satisfy the relationship

$$f_{R}+f_{L}\;=\;f_{ext}$$ (1)

where *f*_*ext*_ is the force applied by the device and *f*_L_ and *f*_R_ are the interaction forces experienced by each participant. This relationship is also enforced by the control law (see above). As noted in the Introduction, there is an infinite number of combinations of interaction forces *f*_R_ and *f*_L_ that satisfy Eq 1.

**Fig 2:**
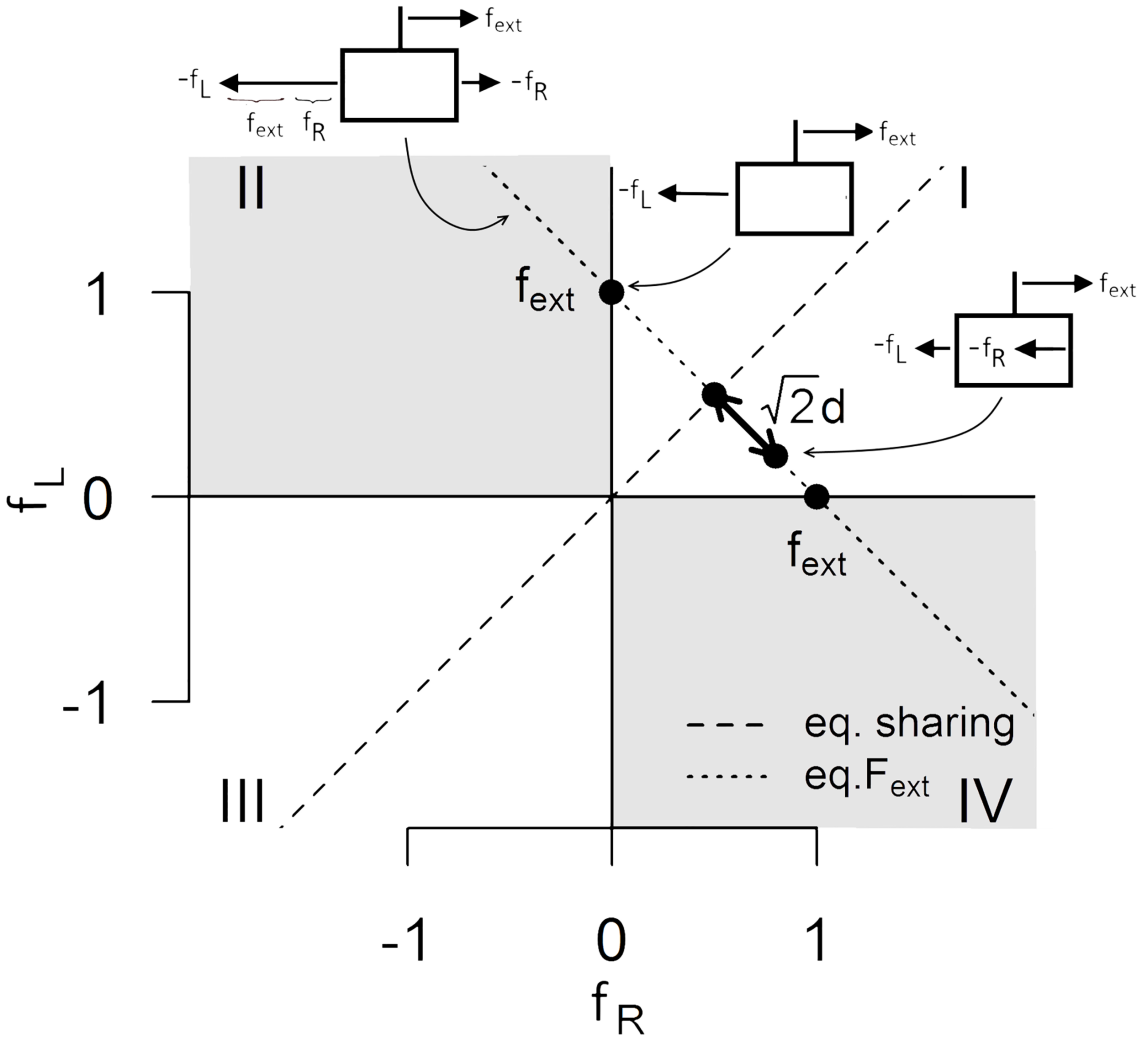
Force sharing strategies. The plane represents all possible combinations of interaction forces (*f*_R_, *f*_L_). The dotted line intersecting the horizontal and vertical axes at *f*_ext._ represents all force combinations corresponding to the same external force *f*_ext_. The intersection of this line with the *f*_R_ = *f*_L_ line is the point at which the two participants split the external force equally. Parameter d is a measure of the distance from equal force sharing. The quadrants highlighted in grey are those in which internal forces are present. *Insets*: The insets illustrate the direction of the forces applied by the two participants (-*f*_R_, and–*f*_*L*_) and the haptic device (*f*_ext_) to the object in the first and second quadrants.

Fig 2 gives a schematic visualization of the relationship between the external force and the corresponding interaction forces. The dotted line represents all possible combinations of interaction forces that balance one external force (*f*_ext_ = 1 N in this example). The insets show three particular combinations of applied forces -*f*_*R*_ and -*f*_*L*_ opposing the external force along this line. The intersections between the dotted line and the vertical (*f*_*R*_ = 0, *f*_*L*_ = *f*_ext_) and horizontal (*f*_*R*_ = f_ext_, *f*_*L*_ = 0) axes correspond to situations where the external force is fully balanced by the left and right participant respectively.

Inside the white quadrants, the two interaction forces have the same sign and both contribute to oppose the external force. The main diagonal (f_L_ = f_R_, see dashed line) represents situations where the force produced (and experienced) by the two participants is the same and corresponds to half the external force, i.e., f_R_ = f_L_ = 0.5f_ext_. We refer to this situation as equal force sharing. Inside the grey quadrants, the signs of the two interaction forces differ. In the second quadrant, the two participants are pulling the end-effector toward them while, in the fourth quadrant, the two participants are pushing against the end-effector. In either case, one of the two participants is producing a force in the same direction as the external force, which must be opposed by his or her partner in addition of the external force. The interaction force in the same direction as the external force together with the component of the other interaction force opposing it are internal forces in the sense that they cancel each other exactly. Such behavior is inefficient from an energetic point of view because the other participant must oppose not only the external force but also the force produced by the partner. It might also be misleading in our task because the interaction force will provide incorrect directional information about the external force to one of the two participants.

In order to summarize the motor behavior in this task with a single parameter, we introduce a force sharing parameter. Because all deviations from equal force sharing must be symmetric for the two participants in order to satisfy Eq 1, we can rewrite f_R_ and f_L_ as:

$$\{\begin{array}{ll} f_{R} & =0.5f_{ext}+d\\ f_{L} & =0.5f_{ext}-d \end{array}$$ (2)

where *d* is the force sharing parameter that specifies how the external force is split by the dyad. Graphically, √2*d* corresponds to the distance between point (*f*_*R*_, *f*_*L*_) and the equal force sharing line along the line corresponding to f_ext_. A positive value of *d* indicates that *f*_R_ > *f*_L_ while a negative value indicates *f*_R_ < *f*_L_. Note that |*d*| < 0.5 *f*_*ext*_ inside the white quadrants and |*d*| > 0.5f_ext_ inside the grey quadrants.

### Optimal force sharing

The interaction force experienced by each participant depends on how the dyad splits the external force. The performance in this task is therefore also likely to be affected by the manner in which the participants split the external force. In this section, we present a simple psychophysical model to find out how the dyad should split the external force to maximize the joint probability that they correctly identify the direction of an external force. We used this model to define the optimal force sharing for each dyad.

In principle, the individual performance is maximized when a participant experiences the full external force, but the task constraints (Eq 1) do not allow the two participants to experience the full external force simultaneously: if one participant opposes the external force singlehandedly, the other participant cannot experience any force and should, in principle, respond at chance level. We therefore assume that the two participants try to avoid such a situation and cooperate to perform the task. We also assume that the two participants respond independently (BI condition) and that each participant’s response is only based on the interaction force that he or she perceives (*f*_R_ or *f*_L_). Under these assumptions, the probability that both participants will correctly identify the direction of an external force f_ext_ = ± *f*_0_ is the product of the probability that each participant will correctly identify it:

$$P(both\;correct|{\|f_{ext}\|\;=\;f}_{0})\;=\;P_{R}(correct|{\|f_{ext}\|\;=\;f}_{0})P_{L}(correct|{\|f_{ext}\|\;=\;f}_{0})$$ (3)

where *P*_*R*_(…) and *P*_*L*_(…) are the probabilities that the left and right participants give a correct response given an external force *f*_ext_ = ± *f*_0_ > 0.

We modeled each participant’s sensitivity to the perceived interaction force with a logistic psychometric function that describes the probability of responding “+Y” (i.e. the external force is directed towards the participant on the right side) as a function of the interaction force:

$$P_{i}(response\;=\;"+Y"|f_{i})\;=\;\psi_{i}(f_{i}|{PSE}_{i},\;{DT}_{i})$$ (4)

where *ψ*_i_ is the logistic function for the left or right participant (*i* = R or L); *PSE*_i_ is the participant’s Point of Subjective Equality that characterizes the offset of the psychometric function and *DT*_i_ is the Detection Threshold, which characterizes its slope. A smaller *DT*_i_ indicates a steeper curve, hence a more sensitive participant. For all participants in our experiment, we fitted a psychometric function to his or her responses in the individual conditions and computed the PSE and threshold DT.

The probability of each participant correctly identifying the direction of the external force depends both on force sharing and on the participants’ psychometric functions. After substituting Eq 2 in Eq 4 and Eq 4 in Eq 3, we can rewrite Eq 3 as:

$$\begin{array}{cc} P(both\;correct|\|f_{ext}\|\;=\;f_{0})= & \left(\psi_{R}\left(0.5f_{0}+d_{p}\right)P\left(f_{ext}\;=\;+f_{0}\right)+(1-\psi_{R}\left(-0.5f_{0}+d_{n}\right))P\left(f_{ext}\;=\;-f_{0}\right)\right)\\ & \left(\psi_{L}\;\left(0.5f_{0}-d_{p}\right)P\left(f_{ext}\;=\;+f_{0}\right)+(1-\psi_{L}\left(-0.5f_{0}-d_{n}\right))P\left(f_{ext}\;=\;-f_{0}\right)\right) \end{array}$$

where *d*_*p*_ is the force sharing when *f*_ext_ = +*f*_0_ and *d*_*n*_ is the force sharing when *f*_ext_ = -*f*_0_. Since the psychometric functions *ψ*_*R*_ and *ψ*_*L*_ are known for a given dyad (see above), we can use this equation to compute the effect of force sharing on a dyad’s probability of responding correctly. For all dyads, we identified the force sharing parameters (*d*_p_, *d*_n_) that maximized the probability of both participants responding correctly for each of the force levels used in the experiment. In general, the best possible performance corresponds to a split of the external force in interaction forces that slightly favors the less sensitive member, so that this person experiences a larger share of the external force. In the results, we compare the actual performance and interaction forces with the optimal ones predicted by this model.

### Performance measure

To evaluate the performance of the participants, we computed the probability of correctly identifying the direction of the interaction and external forces for a standard level of force (0.5N). The interaction and external forces are the same in the individual conditions but not in the joint conditions, where the interaction force depends on how the dyad balances the external force. To compute this probability, we fitted the individual responses of each participant as a function of the magnitude of the interaction or external force with an exponential function:

$$P(correct|f)\;=\;\alpha +(1-\alpha )(1-e^{-bf})$$ (5)

where *b* is a free parameter that corresponds to the slope of the exponential curve, that is how fast the percentage of correct responses increases as a function of the force, and α = 0.5 is the chance level. Then, we estimated this function for *f* = 0.5 N, where *f* could be the interaction or external force. This procedure is necessary because it is not possible to estimate the participant’s performance for a specific level of interaction force in the joint conditions since the experimenter does not control the interaction forces in the joint conditions. Moreover, it also allows one to compare the performance between the two groups of dyads, which were tested with different levels of external force (see Table 1).

## Results

### General behavior

Table 2 reports general characteristics of the participants’ behavior in the four experimental conditions. Thirty-seven trials (0.8%) were removed from the analyses because the participant responded before the auditory cue that signaled the end of the ramping period. The end-effector movements from the beginning of the ramp period until the end of the trial were in general below 1 cm. The median ± MAD response time was 0.61 ± 0.34 sec in the independent response conditions (L, R and BI) and 1.25 ± 1.17 sec in the negotiated response condition (BN). In this latter condition, the dyads agreed on the first response in 80.5% of the trials, on the second in 13.3% and on the third in 3.8%. Only 2.4% of the trials required a larger number of responses to reach an agreement.

**Table 2:** General characteristics of the of the participants’ behavior.

	Duration [s]	Path length [cm]	Within-trial force-sharing SD [N]	Between-trial force-sharing SD [N]	Point of Subjective Equality [N]	Differential Threshold [N]
L	5.65±0.40	0.61±0.30	-	-	0.02±0.11	0.12±0.07
R	5.63±0.38	0.60±0.29	-	-	-0.04±0.07	0.10±0.03
BI	5.83±0.55	0.58±0.23	0.073±0.051	0.36±0.13	-	-
BN	6.25±1.17	0.67±0.35	0.092±0.070	0.32±0.12	-	-

The data are reported as median ± median absolute deviation (MAD).

**Fig 3:**
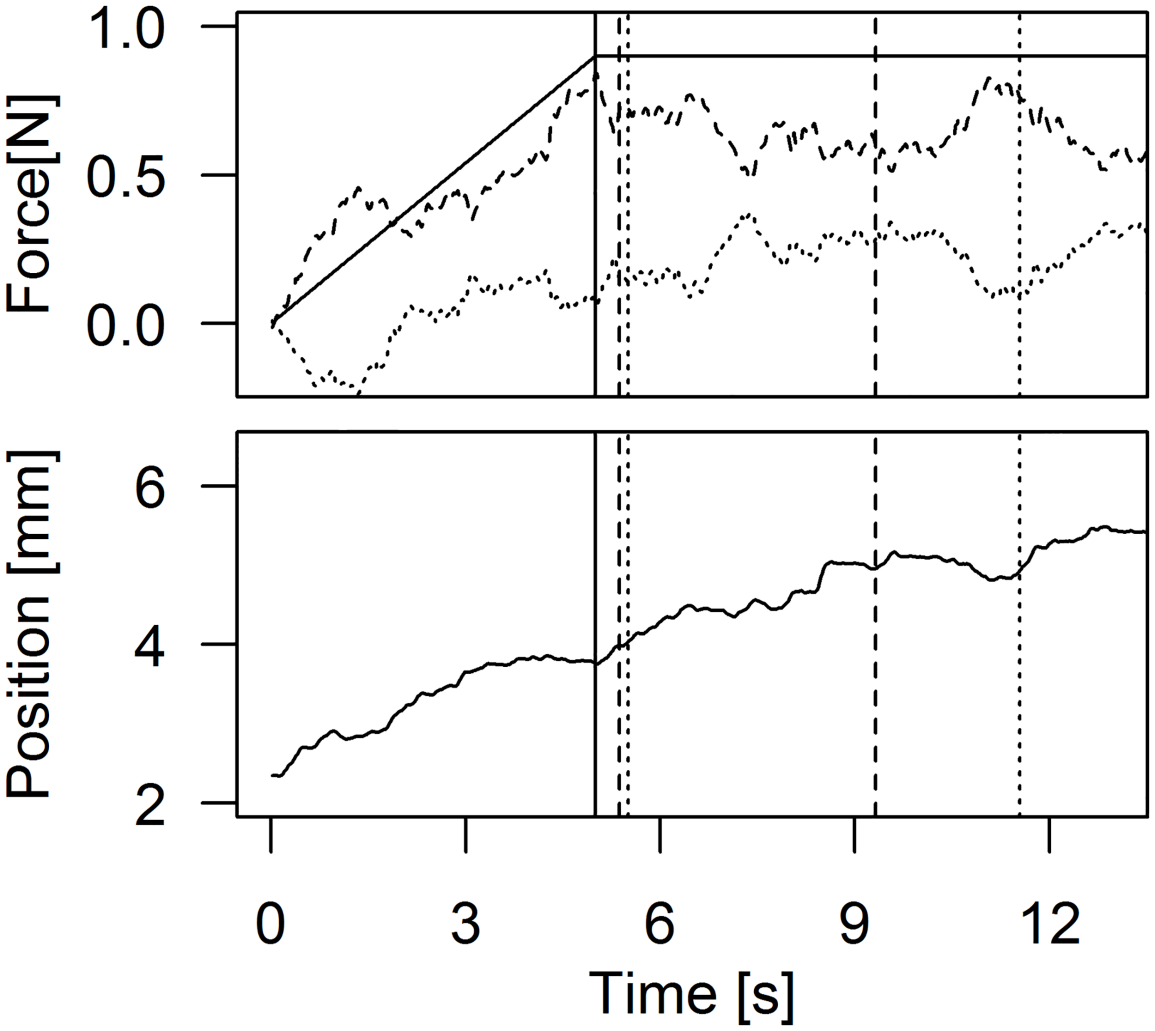
Example of force and position trajectories from a trial. *Top*: Interaction forces of the right (dashed) and left (dotted) participants during a joint trial with negotiated response. The sum of the two interaction forces corresponds to the external force (0.9 N, solid line). The within-trial variability of the interaction force is 0.08 N. *Bottom*: Position of the end-effector during the trial. In both plots the solid vertical lines indicate the end of the ramp period. Each participant responded twice in this negotiated trial (the dashed and dotted vertical lines indicate the time at which each participant responded). The path length for this trial is 5.3 mm.

In the joint conditions, the value of the force sharing parameter was computed for each sample during the trial. The value of force sharing (d) was relatively stable within each trial (see within trial force sharing SD in Table 2). For this reason, we computed the average value across time for each trial and also computed its variability across trials (see between-trial force-sharing SD in Table 2). Fig 3 shows a representative example of a trial in the negotiated condition.

The PSEs and DTs of the logistic psychometric functions computed from the individual trials were in line with those reported in [19]. A more detailed analysis of the PSEs and DTs can be found in [20].

### Individual performance

**Fig 4:**
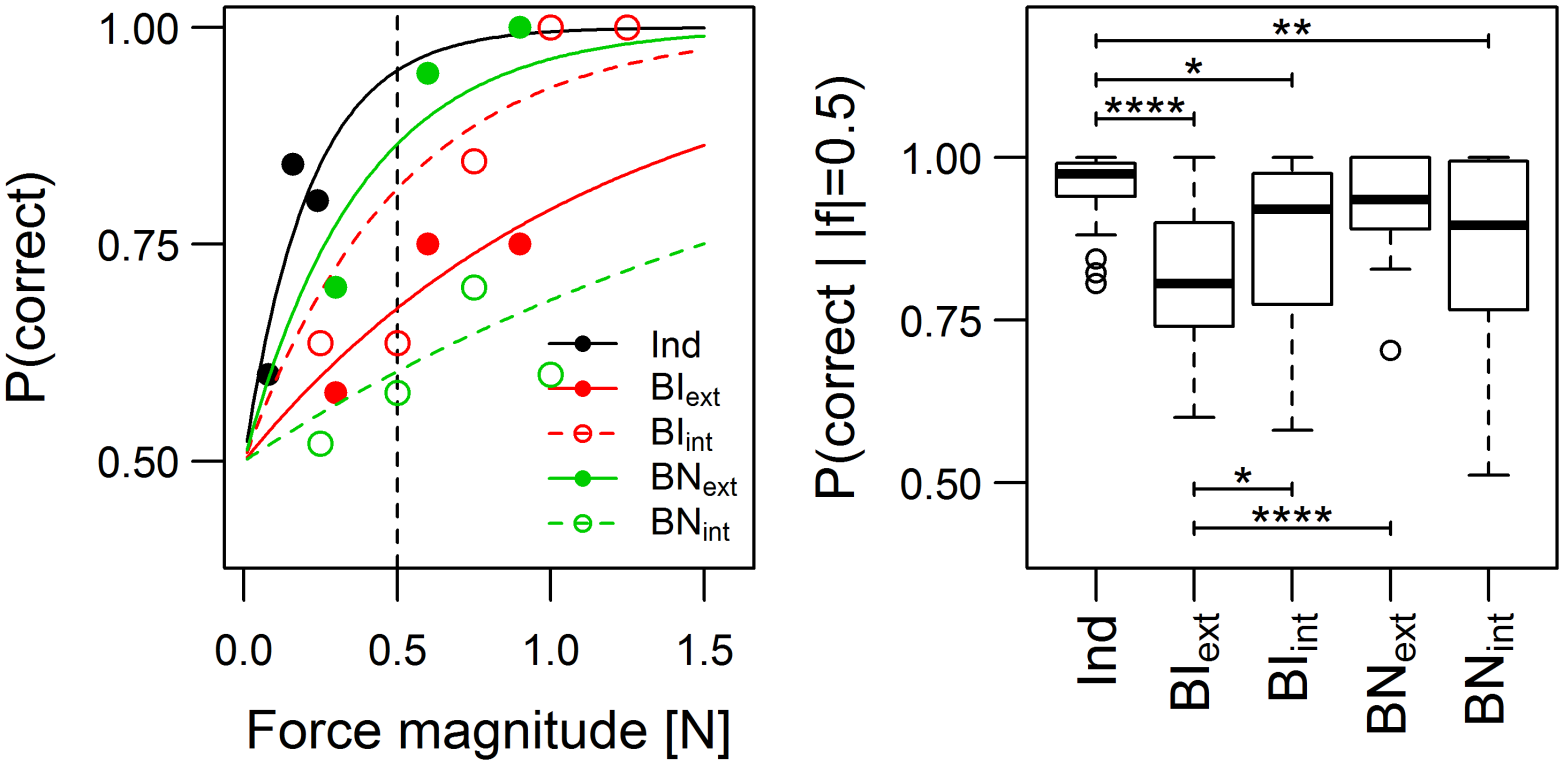
Individual probability of correct response. *Left*: Probability of correct response as a function of the force magnitude (interaction force or external force) for one participant in all conditions. Probability curves correspond to Eq 5. *Right*: Probability of participants correctly identifying the direction of a force of 0.5 N in the individual (Ind) and joint conditions. For the joint conditions, both the probability of identifying the direction of the external force (BI_ext_, BN_ext_), and the probability of identifying the direction of the interaction force (BI_int_, BN_int_) are shown. * p<0.05; ** p<0.01; *** p<0.001; **** p<0.0001.

Fig 4 (left panel) shows the probability of correctly identifying the direction of the interaction or external force as a function of its magnitude for one participant in all conditions. In the joint conditions, the data points for the interaction force (empty circles, BI_int_ and BN_int_) correspond to the proportion of correct responses for trials with interaction force in 0.25 N bins ranging from 0 to 1.5 N. The right panel of Fig 4 compares the probability of correctly identifying the force direction across conditions for all participants. For the joint conditions, BI_ext_ and BN_ext_ refer to the probability of correctly identifying the direction of a 0.5 N external force, while BI_int_ and BN_int_ refer to the probability of correctly identifying the direction of a 0.5 N interaction force, in the same conditions.

The difference between groups was statistically significant as assessed by a non-parametric Friedman rank sum test (χ^2^(4) = 43.77, p<0.001). We performed pairwise comparisons between groups using a non-parametric rank-based multiple comparisons test (see [21]). Analyzing the coefficients of the exponential fits instead of the performance at 0.5N yields the same results.

The performance in the individual condition (Ind) was significantly higher than that obtained in all other conditions except BN_ext_. The poorer performance in BI_ext_ with respect to the individual condition *Ind* can be attributed to the fact that, in the joint conditions, the participants did not experience the full external force in general. In these conditions, they experienced the interaction force, which depended in part on what their partner was doing (see next section). The difference between Ind and BI_int_ is more surprising, since one would expect that the performance in BI_int_ should be the same as in the individual condition if the participants based their responses on the interaction force. A part of this difference could be attributed to the increased variability of the interaction force in BI_int_ relative to Ind, even though this variability was found to be small.

### Force sharing

For each trial in the joint conditions, we computed the average value of the force sharing parameter *d*. Then, we computed the across-trial average and standard deviation. As shown in Table 2, the within-trial variability of force sharing was relatively low, which indicates that interaction force remains relatively stable during a trial. In contrast, the force sharing SD across trials with the same external force was relatively large, far larger than the SD within the individual trials. This indicates that dyads varied the way they split the external force across different trials.

**Fig 5:**
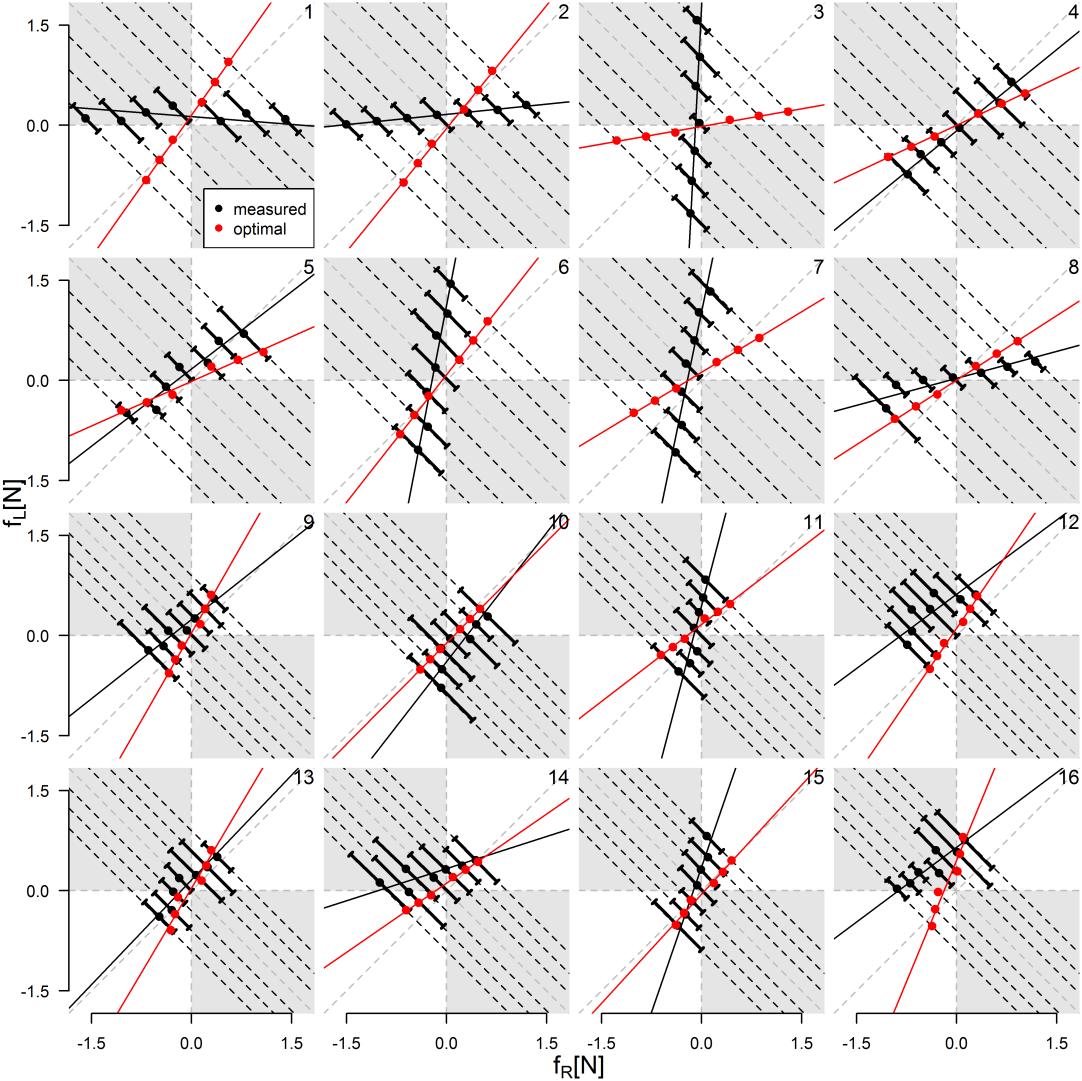
Average ± SD force sharing and linear regression best fit in BI condition. Measured results are displayed in black and optimal force sharing in red. The grey areas indicate the presence of an internal force.

Fig 5 shows how the interaction force and force sharing parameter varied as a function of the external force for all dyads. The first observation is that force sharing in the dyad is highly idiosyncratic. While some dyads share the force equally (e.g. dyad 4), in others there is a marked prevalence of one participant (e.g. the right participant in dyad 1, or the left one in dyad 3) absorbing all the force, while the partner experiences a very low interaction force. Another strategy observed (e.g. in dyad 16), is one where average force sharing is distributed parallel to equal force sharing but with an offset. This represents a case in which the dyad shares the force equally, but with an added internal force (participants pushing or pulling against each other).

Despite this marked variability, we see that in all dyads but one (dyad 12), the average force sharing values across trials (black dots) vary on average in an orderly manner across the different levels of the external force. This indicates that there is a degree of regularity in the dyads’ behavior. To describe this tendency synthetically, we computed the regression line that best fitted the average force-sharing values for each dyad (black line).

For each dyad, we computed the force sharing that maximized the probability of both participants correctly identifying the direction of the external force (optimal force sharing), as explained in the Data Analysis and Modeling section. The red dots in Fig 5 indicate the computed optimal force sharing for each dyad and external force level. While the model often predicts the optimal force sharing to be close to the equal force sharing, there are notable exceptions such as dyad 3 for whom the best strategy would be for the right participant to absorb most of the force. These deviations from equal force sharing are due to differences in the participants’ sensitivity. The model, indeed, attributes a larger share of the force to the less sensitive participant (in dyad 3 we have DT_R_ = 0.15N, DT_L_ = 0.02N). Many dyads did not adopt optimal force sharing (see for example dyads 1, 2, 3 and 7).

**Fig 6:**
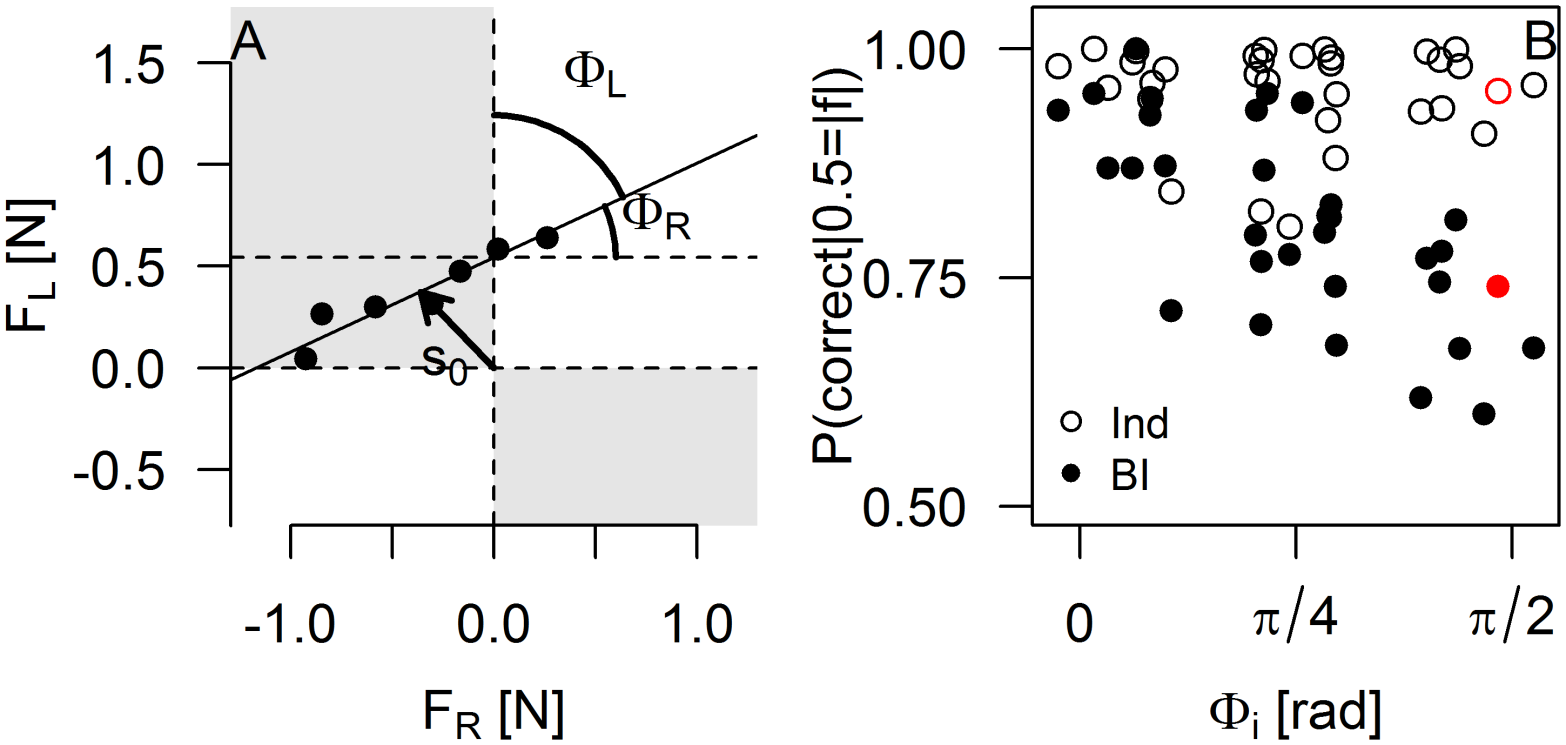
Probability of correct response in the BI condition for a 0.5N external force as a function of force sharing for all participants. A: Parameters used to describe the dyads’ force sharing. B: Probability of correct response in the BI condition as a function of Φ_R_ and Φ_L_. The participants’ probability of correct response in the individual conditions is also plotted for comparison. Participant 3R, shown in Fig 7, is highlited in red in B.

In principle, force sharing should have a large impact on performance. To take an extreme example, one would expect that the participant taking most of the external force would perform much better than the one that takes no force and has no information about the external force. Another question is whether dyads use the internal force to improve their performance. In order to quantify the relationship between the force sharing adopted by the dyads and their performance in the BI condition, we used the following two parameters that characterize the regression lines of Fig 5. The first parameter is the offset s_0_ that describes the internal force that exists in the absence of an external force. The second parameter is the angle *φ*_i_ which indicates how much external force each participant is taking as shown in Fig 6A (the angle is with respect to the vertical for S_L_ and with respect to the horizontal line for S_R_). A null value *φ*_i_ = 0 indicates that the participant is taking all the external force, while *φ*_i_ = π/2 indicates that the participant experiences no interaction force. Equal force sharing between the participants gives *φ*_R_ = *φ*_L_ = π/4.

Fig 6B shows participants’ performance in the BI task as a function of the angular force sharing *φ*_i_, which indicates how much external force each participant experiences. As expected, there is a strong negative correlation between the two variables (*r* = -0.70). Participants who absorb all the external force (i.e. *φ*_i_ ≈ 0) perform as well as in the individual condition, while participants who receive a smaller share of the external force perform less well. While our model predicted a relationship between the participants’ force detection threshold (DT) and the angular force sharing *φ*_i_, with the less sensitive participant receiving more force, we found no such relationship for the force sharing adopted by the dyads (*r* = 0.13).

While the correlation in Fig 6B is expected, the figure also indicates that participants with *φ*_i_ ≈ π/2 perform surprisingly well. Because of their force sharing, these participants experienced an interaction force close to zero, and would therefore be expected to perform close to chance level. Fig 6B indicates that their performance is lower than that of the other participants, but well above chance.

### Position-based response

The surprisingly good performance of participants receiving no force needs an explanation, because it appears a priori impossible. If the interaction force provides no cues, the only other possible source of information is the position or movement of the device. While dyads were instructed to keep the device at its initial position, we could still observe a small movement (see path length in Table 2). Interestingly, this residual movement was correlated with the direction of the external force in the BI condition. The average ± SD correlation across dyads between the net movement *x*end—*x*_start_ and the external force *f*_ext_ was 0.78 ± 0.08.

**Fig 7:**
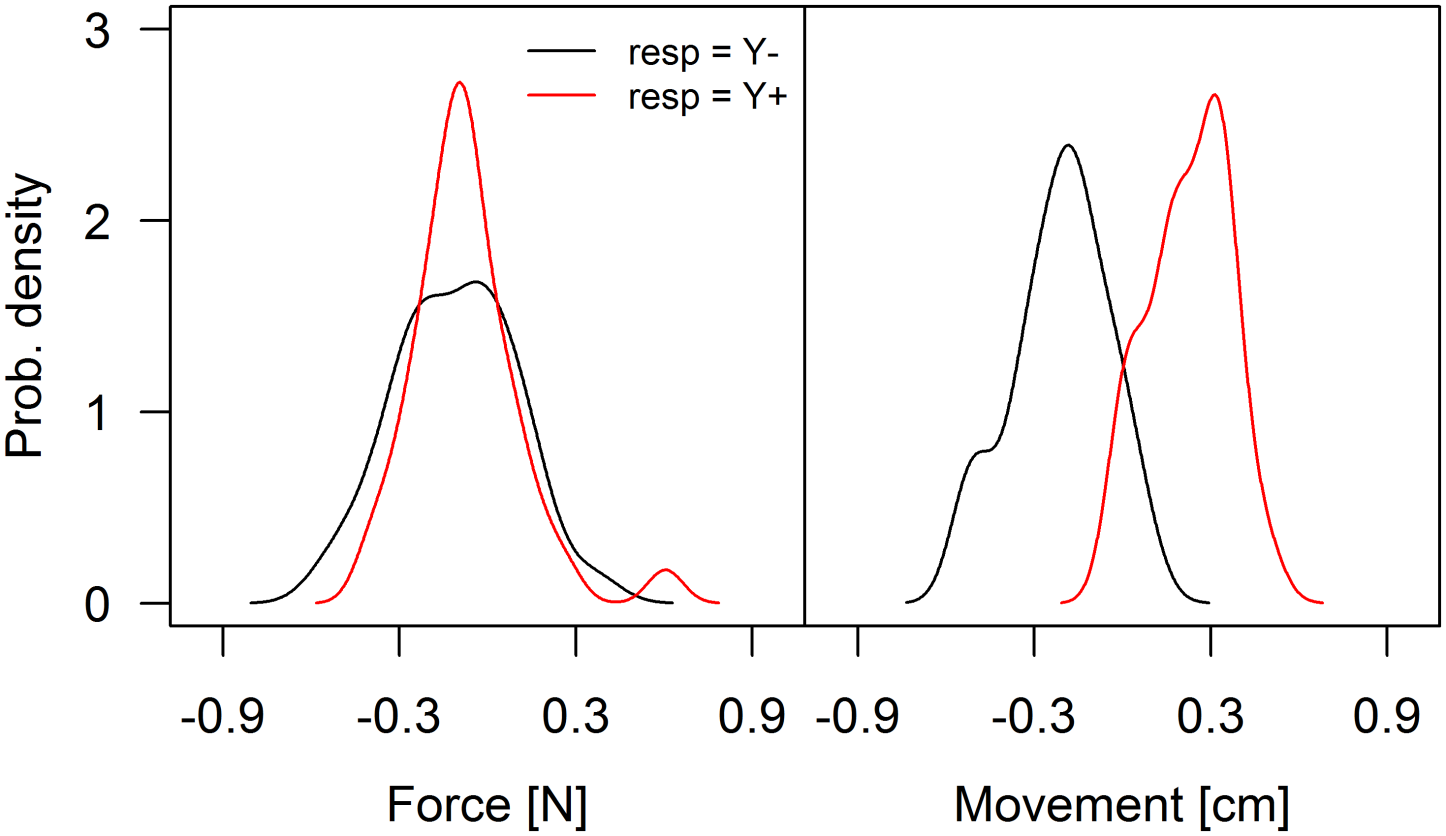
Example of probability density functions of interaction force and hand movement. The plots refer to participant 3R. The distributions displayed in black and red relate to the trials in which the participant indicated that the external force was directed towards one or the other direction.

To further investigate the contribution of force and position cues to the response of the participant, we compared the distribution of the responses as a function of the interaction force and hand movement in the BI condition. Fig 7 (left panel) shows the distributions of the two responses as a function of the interaction force for participant 3R who, on average, experiences no force (force sharing *φ*_R_ is 1.52 rad). The two distributions overlap and are centered on the zero force, indicating that the responses are independent from the interaction force. In contrast, the distributions of the two possible responses are much better separated when they are associated with the hand movements (right panel). To quantify the density curves’ separation, we computed a d’ index for all participants as follows:

$$d^{\prime }\;=\;\frac{\mu_{Y-}-\mu_{Y+}}{\sqrt{\frac{1}{2}(\sigma_{Y-}^{2}+\sigma_{Y+}^{2})}}$$

**Fig 8:**
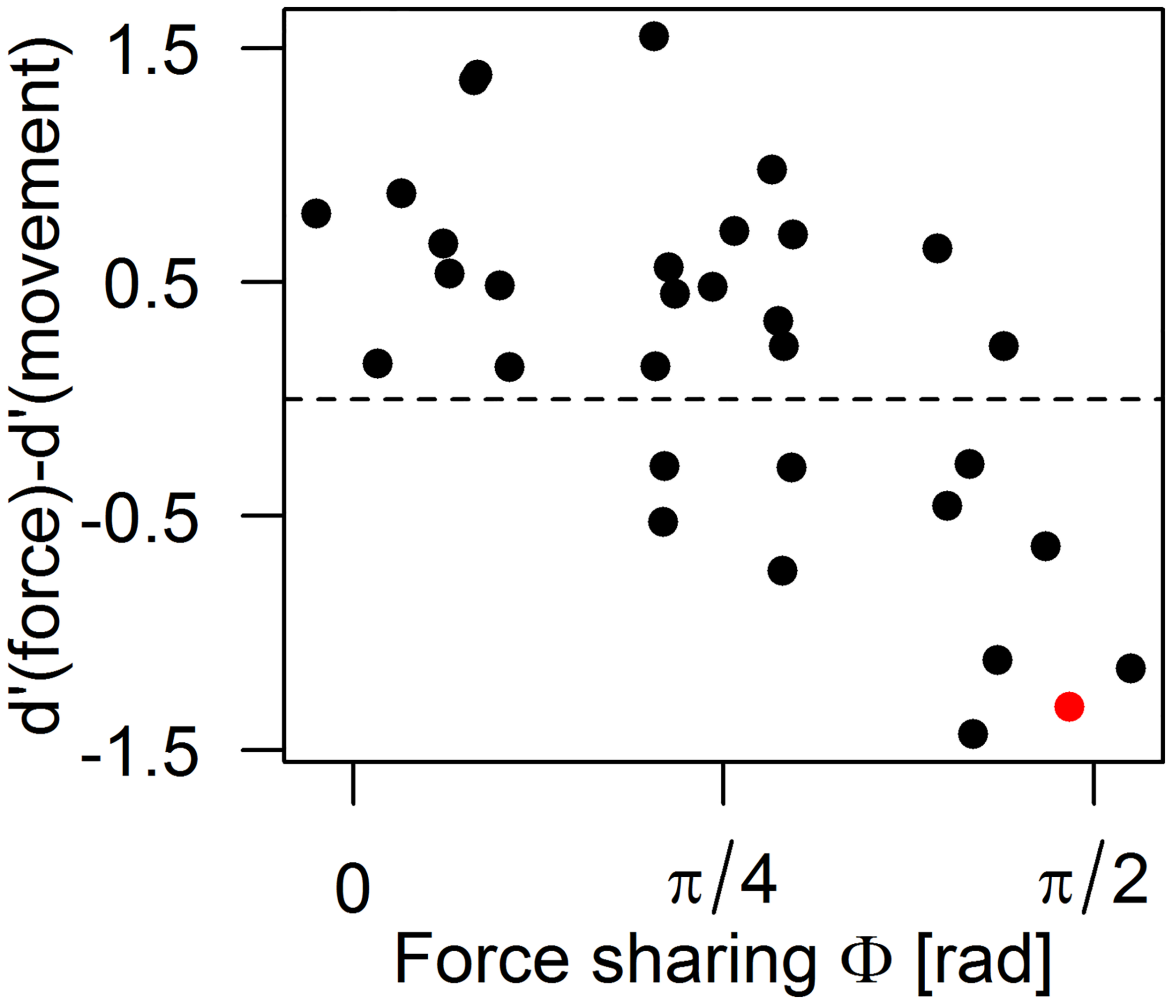
Difference between force and movement *d’* as a function of force sharing for all participants. Participant 3R, shown in Fig 7, is highlighted in red.

Fig 8 shows that the difference *d’*(force)—*d’*(movement) is negatively correlated with the amount of interaction force experienced by a participant (*r* = -0.66). In other words, it shows that the responses provided by participants who are penalized by the force sharing are explained better by their hand movement than by the interaction force they experience.

### Predicted and measured dyadic performance

**Fig 9:**
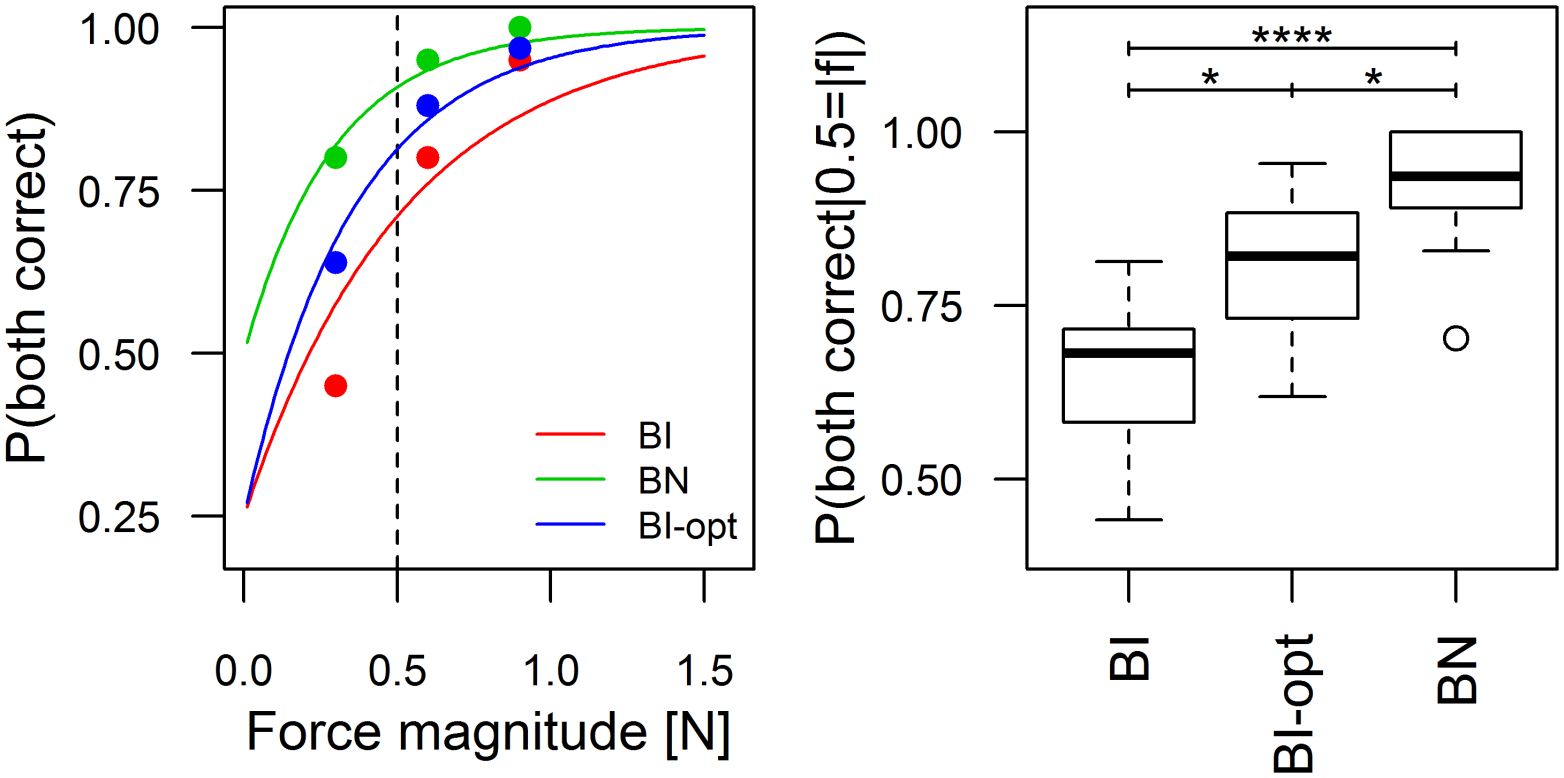
Dyadic probability of correct response. *Left*: Probability of both participants responding correctly as a function of the force magnitude for one dyad in all joint conditions (BI and BN). The prediction of the model for optimal force sharing is also included (BI-opt). *Right*: Probability of both participants correctly identifying the direction of the external force. We show the results measured in the BI and BN conditions and those predicted by the model for optimal force sharing (BI-opt). * p<0.05; ** p<0.01; *** p<0.001; **** p<0.0001.

For all dyads and levels of external force in the BI and BN conditions, we computed the proportion of trials where both participants responded correctly. We then fitted the exponential function of Eq 5 assuming a chance level α = 0.25 in the BI condition, where the two participants respond independently, and *α* = 0.5 in the BN condition, where the two participants had to agree on the response. Fig 9, left shows an example of the curves obtained for one dyad. This figure also includes the probability that both participants would respond correctly for optimal force sharing. From these curves, we computed the probability that both participants would respond correctly when the external force is 0.5 N. Fig 9, right shows the results.

We tested the statistical significance of the difference between groups for the results in Fig 9, right with a non-parametric Friedman rank sum test (χ^2^(2) = 28.125, p<0.001) and performed pairwise comparisons between groups using a non-parametric rank-based multiple comparisons test (see [21]). We found that the probability of both participants correctly identifying the direction of the external force in the independent-response (BI) condition was significantly lower than that predicted for optimal force sharing. When participants negotiate the response, their performance improves significantly over the BI condition and even exceeds the performance predicted by the model for optimal force sharing.

**Fig 10:**
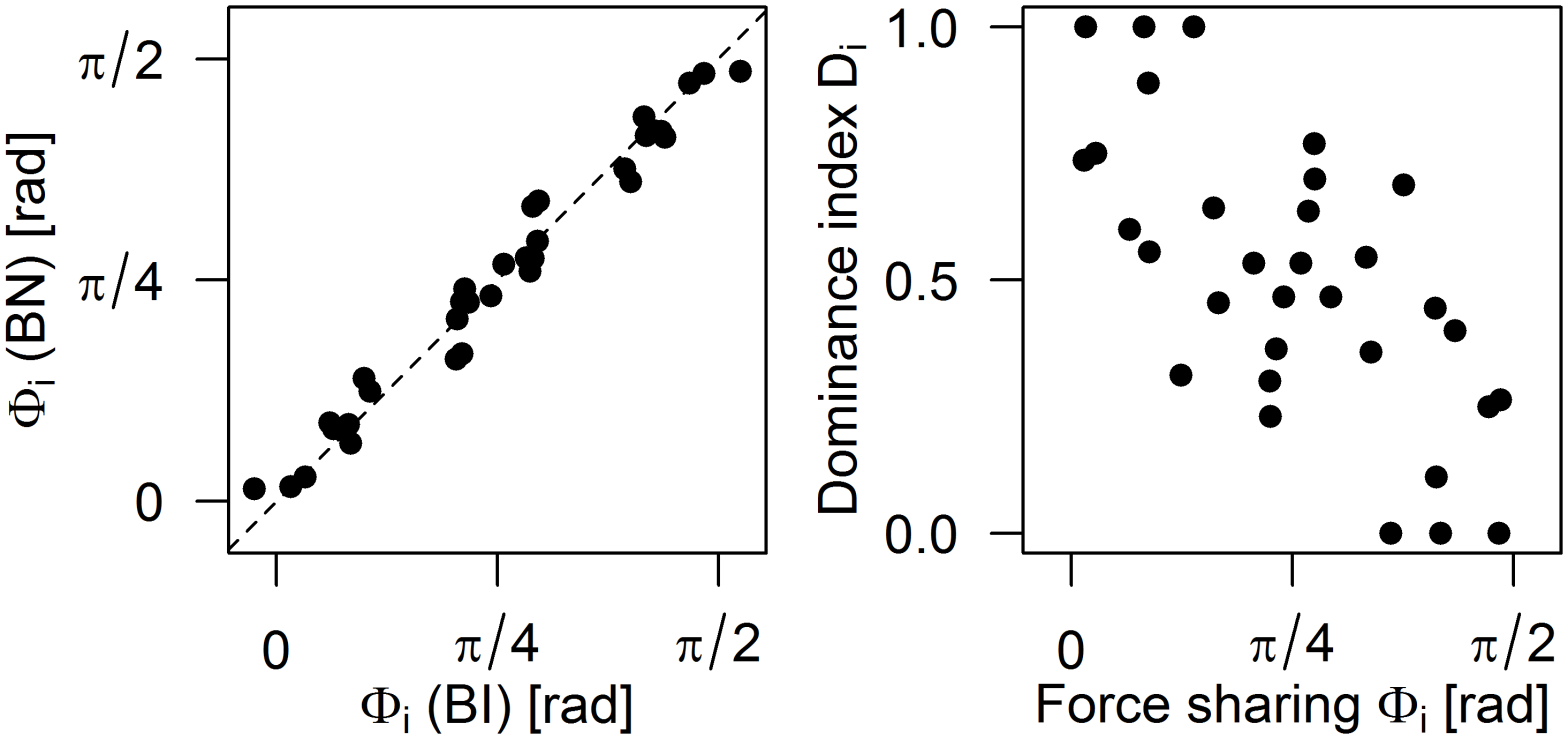
Force sharing and the dominance index in the negotiated condition. *Left*: Comparison between force sharing in the BI and BN condition for all participants (*i* = L or R depending on the participant). *Right*: The dominance index as a function of force sharing in the BN condition for all participants.

The first hypothesis as to why dyads perform better in this condition could be that the negotiation process leads them to adopt force sharing that is closer to optimal. However we found that this was not the case. The force sharing parameters φ_i_ were highly correlated in the BI and BN conditions (r = 0.988, Fig 10, left)). The average ± SD absolute difference |φ_i_(BI)- φ_i_(BN)| was 0.06 ± 0.05 rad, which is not statistically different from zero. The average ± SD absolute difference |S_0_(BI)-S_0_(BN)| was 0.06 ± 0.06 N, indicating that dyads adopted the same force sharing in BI and BN.

The second hypothesis is that the improvement in performance might be due to the fact that the participants who perceived no force due to the force sharing they adopted could choose to agree with their partner regardless of the perceived force in the BN condition. To test this, we computed a dominance index *D*_*i*_ for each participant, which we defined as the fraction of trials, among those where the dyad initially disagreed, in which the participant determined the final response (i.e. the participant did not change his/her initial response). This index takes values between 0 (the final response was always determined by the other participant) and 1 (the participant always determined the final response) and for each dyad we have *D*_*R*_ + *D*_*L*_ = 1. The distance |*D*_*L*_*—D*_*R*_| quantifies the imbalance in dominance. For example, if one participant determined all the responses the distance would be 1 (*D*_*L*_ = 1, *D*_*R*_ = 0, or the opposite), while if each participant determined half of the responses the distance would be 0 (*D*_*L*_ = *D*_*R*_ = 0.5). The average ± SD value of this distance across all dyads was 0.75 ± 0.50, indicating an average tendency of dyads to put one of the two participants in charge of determining the response, with the other following the lead. This strategy is efficient if the participant who determines the response is the one favored by force sharing. To test for this, we compared the dominance index to force sharing and found a strong negative correlation (*r* = -0.69, see Fig 10, right). This result indicates that indeed participants who take the larger share of the external force tend to also dominate the response, while participants with unfavorable force sharing tend to follow their partner’s response. While we found this strong correlation between the dominance index and force sharing, the correlation between the dominance index and individual participants’ force detection threshold was weak (*r* = -0.33) indicating that the latter is a less relevant factor than force sharing in the negotiated condition.

## Discussion

In our study we investigated how two people perceive a force applied to an object that is held jointly. This task requires that they untangle the different force components that contribute to the interaction force, namely the component due to the partner’s actions and the external force applied by the haptic device to the object. A priori, this task might appear impossible because the interaction force experienced by each participant combines the two other components in an irremediable way. However, our results clearly show that participants responded well above chance level even though their performance in identifying the direction of the external force was less accurate than when they performed the task alone.

The first element of response is the observation that, despite considerable trial-to-trial variability, the external force was split in an orderly (linear) manner when considering the average interaction force values. To a first approximation, the external force tended to be split according to a fixed ratio (the angle φ_i_). Another interesting observation is that this ratio could vary markedly across dyads. While some dyads split the external force approximately equally, others did not. This implies that one of the two people did not receive relevant force information about the external force. The implications of these two findings are discussed in more detail below.

**Fig 11:**
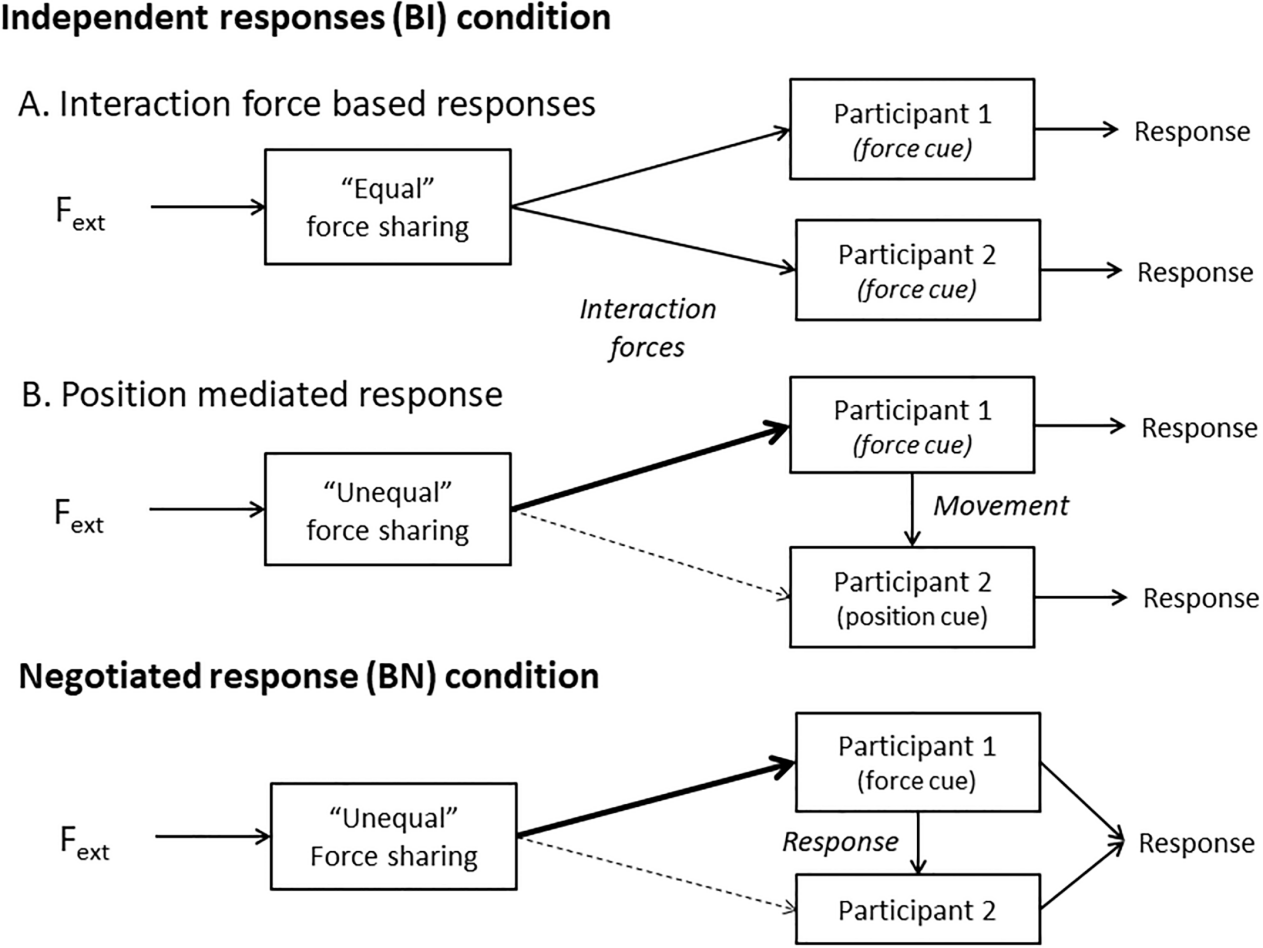
Response strategies in joint force perception tasks. *A*: the external force is approximately equally split between the two participants. Both use the corresponding interaction force to respond independently. *B* and *C*: the *thick* and *dashed arrows* represent the strong and weak interaction force in a dyad where the external force is split unequally. In both cases, participant 1 balances the whole external force while participant 2 experiences almost no force.

The observation that the external force tends to be split in an orderly manner is important because it shows that dyads’ motor behavior is independent from the value of the external force. As noted above, we also found that the proportion of external force that each person opposed varied considerably across dyads. In fact, our results suggest that different dyads used different cues and strategies to perform this task (see Fig 11).

### Force sharing: Significance, optimality and emergence

One possible strategy is to use the interaction force as the basis to decide the direction of the external force (see interaction force based responses in Fig 11). This strategy works for the two participants only if they coordinate their motor behavior so the directions of the two interaction forces reflect the direction of the external force. In other words, the dyads must avoid producing internal forces in this case. In addition, it seems necessary for the two participants to split the external force approximately equally, so that both can perceive the interaction forces. As a matter of fact, if the external force were split very unequally and one participant balanced the external force singlehandedly, the other participant would not experience any interaction force and would have to respond at chance level. To address this issue more precisely, we computed the force sharing that would maximize the joint performance of the dyad based on the discrimination capacity of each participant measured in the independent conditions. Our results clearly show that only some dyads behaved in a way that corresponds to this optimal force-sharing model. As a matter of fact, for many dyads, the external force was split very unequally, with one participant opposing the external force and the other participant experiencing no force on average (see next section).

Another question is how the dyads might coordinate their motor behavior so that the interaction forces reflect the external force given that neither participant knows what the external force is or what the other participant is doing. While our study was not designed to address this question, a possibility is that both members of the dyads behaved like springs:

$$f_{ext}=f_{R}+f_{L}=k_{R}\left(x-x_{R}\right)+k_{L}\left(x-x_{L}\right)$$

where *x* is the position of the object, *k*_*R*_ and *k*_*L*_ are the stiffnesses of the participants, *x*_*R*_ and *x*_*L*_ are the reference positions. Both the stiffnesses and reference positions are under the control of the dyad members, who experience the corresponding interaction forces. Assuming that the control variables do not change, this simple model will split the external force according to a ratio that depends on the stiffness chosen by the dyad members. While this model presents analogies with the Equilibrium Point Hypothesis [22], it is unlikely that the stiffness considered here corresponds to the passive properties of the musculoskeletal systems of the two participants. The interaction forces are too small to have any significant mechanical effect besides stimulating the mechanoreceptors of the fingertips. It is much more likely that the interaction forces result from the way the dyad reacted to the tactile and proprioceptive signals, such as holding the hand position or minimizing the interaction force. It is important to note that this strategy requires cooperation and coordination for the dyad to behave as springs and avoid producing internal forces.

For dyads where one participant experienced the full external force while the other participant experienced no force, the behavior of the two participants in the dyad might correspond to that of an impedance-type controller minimizing sensed displacement versus an admittance-type controller minimizing sensed force. While these ideas need further confirmation, the main point here is that force sharing does not necessarily require the two participants to know what the external force is. Instead, force sharing might emerge from the interaction between the motor behaviors of the two participants.

### Position-mediated force perception

A surprising observation was the unexpectedly good performance of participants who did not get any force on average in the dyad. In the absence of interaction force, one would expect the rate of correct responses to be at chance level. While these participants’ performance was less accurate than that of the participants who experienced all the force, it was clearly above chance level. For these dyads, we found that the participants who took no interaction force used a position cue to respond (see position-mediated response in Fig 11). This strategy requires that the participant who has the force information moves the device according to the external force. This movement provides the crucial bit of information to the participant without force information. It is unlikely that this movement occurs passively because the external forces used in our experiments are very small compared to the weight of the hand and forearm and are therefore unlikely to move the device and arms of the participants. This strategy can be seen as a form of implicit haptic communication where one participant receives full information on the external force and communicates it to the partner by moving the device in the appropriate direction. Interestingly, the two members of the dyad are apparently not conscious of using such a strategy as nobody reported it at the end of the experiment when asked what strategy they used in the task.

A parallel can perhaps be drawn with the control of joint action in motor tasks involving physical interaction where the two people in the dyad assume different but complementary roles. For example, previous reports have revealed specialized strategies in reaching tasks with a dual-handle crank where one participant controls the crank’s acceleration and the partner controls the crank’s final position, either by applying an opposing force [23] or by applying a tangential force and increasing the stiffness of the system [24]. These results have been interpreted in terms of one partner leading the other partner in these motor tasks. Our study shows similar role dissociation in a perception task where, for some dyads, one person might provide the information necessary for the other to respond.

### Internal forces and other possible response strategies

A priori, other response strategies might have been used to perform this task. For example, research in joint motor tasks suggest that internal forces represent an information channel that plays an important role in coordinating action (e.g. [2,25]). For example, it has been observed that internal forces increase with coordination requirements in motor tasks where two people need to move an object together [2]. While there was considerable between-trial variability in our study the interaction forces had the same sign on average, which indicates that participants tended not to produce internal forces in this task. Internal forces were absent in more than 50% of the trials and, when present, their magnitude was quite small (0.2±0.05 N on average). Contrary to what would be expected if internal forces played an important informative role in this task, the amount of internal force produced by a dyad was not correlated with the dyads’ performance.

Other studies have emphasized the importance of having shared representation and models to predict the effects of one’s own and others’ actions [1]. In our study, the task could be performed in principle if both dyad members had an internal model that allowed them to predict the force produced by the other. However, it is difficult to see how participants might acquire such a model because the interaction forces provide highly ambiguous and not very helpful information in this task. This difficulty is compounded by the fact that the external force varied randomly between trials. This is a significant impediment for the development of an internal model of the others’ actions because the environment randomness brings uncertainty about what the other person is doing. This situation is very different from more classic motor learning studies where the two participants must coordinate their action and develop an internal model of the environment or object dynamics to perform a sensory-motor task. In the latter case, the properties of the object or the environment usually remain the same during the experiment [26,27]. Future studies are needed to understand whether it is possible to develop a useful internal model of the others’ actions in contexts similar to the one in our study.

### Negotiating the response and explicit communication

The negotiated condition was introduced in the study to see whether having the possibility to communicate and agree on the response would change the way the external force is split. Such an effect could provide indirect information about how interaction forces are used when the dyad cannot communicate. This was not however the case: while we observed great improvement in the performance in this negotiated-response condition, we did not observe any significant change in force sharing with respect to condition where each participant would respond independently. Noticeably, this performance gain also occurred in the dyads where one participant experienced no force. In this case, we found that the participant who experienced the least force was much more likely to switch his response than the participant who experienced the most force (see dominance-mediated response in Fig 11).

The fact that participants who took the largest share of external force also tended to maintain their response during the negotiation phase while the other participants tended to follow their partner’s response is an interesting observation because force sharing and negotiation refer to different and possibly independent aspects of the dyad’s behavior. That said, the observation that the readiness to follow the other person depends on the level of force experienced makes sense since the participant who does not experience much force cannot have a high level of confidence in his or her response. Moreover, this readiness to follow the partner’s response in the negotiated response (BN) condition is akin to the readiness to follow the partner’s movement in the independent response (BI) condition. In both cases, the participant without force information relied on the partner to respond.

## Conclusion

Altogether, our study reports on participants’ ability to untangle the forces at play in a joint manipulation task. Importantly, we found that dyads used different strategies to perform this task depending on how the force was split between the two people. Despite considerable between-trial variability, we believe that the tendency to split the force in a manner approximately proportional to the external force is an important observation to explain how these dyads performed this task. Importantly, all these strategies imply some form of coordination. Here, we submit that many dyads adopted a motor behavior whereby the external force was at least partially reflected in the interaction force (force sharing response strategy). Adopting such a motor behavior is a form of joint action because it requires that the two participants cooperate when interacting physically. We also highlighted another possible response strategy, whereby one participant communicates information on the perceived force to his/her partner by changing the position of the device (position-mediated response strategy). This latter strategy illustrates how members of a dyad might communicate haptically when one partner does not have access to relevant information. This form of joint action reflects an asymmetry in the available information and a corresponding specialization of the role of each partner. Whether the asymmetry in force sharing is the cause or effect of adopting such a specialized response strategy is an open question. In any case, the presence of these two strategies, which are not necessarily mutually exclusive, shows it is important to remember that both position and force can be used to transmit information, even in a force perception task where position seems a priori irrelevant.
